# Tipping the scales: a theoretical model to describe the differential effects of the COVID-19 pandemic on mortality

**DOI:** 10.1186/s12939-021-01470-x

**Published:** 2021-06-16

**Authors:** Mor Saban, Vicki Myers, Osnat Luxenburg, Rachel Wilf-Miron

**Affiliations:** 1 Gertner Institute for Epidemiology and Health Policy Research, Sheba Medical Center, Ramat-Gan, Israel; 2 Medical Technology, Health Information and Research Directorate, Ministry of Health, Jerusalem, Israel; 3 School of Public Health, Sackler Faculty of Medicine, Tel Aviv University, Sackler, Israel

**Keywords:** Covid-19, Pandemic, Model, Morality, Resilience, Health behavior

## Abstract

**Background:**

The COVID-19 pandemic has resulted in changes in almost every aspect of life. The fatal consequences of the pandemic have been clearly reported, with direct and indirect effects; however, there is some evidence of a positive secondary impact, such as fewer motor accidents, lower influenza burden and reduced air pollution.

**Methods/model:**

We present a model to describe the differing effects of the COVID-19 pandemic on mortality, taking into account external pressures and internal resources and their relationship with resilience and health behaviors, which affect mortality risk, inspired by elements of the salutogenic model.

Individuals with lower resources and from more deprived communities are likely to be more negatively affected by the external changes occurring, while those with more resources may be more likely to experience the benefits. Both individual and community resources affect coping and influence both mental and physical health.

**Conclusions:**

Decision makers should consider ways to incorporate the positive changes which occurred as part of the exit strategy. Societies should invest in building resources to improve both individual and community resilience to help people be better prepared and more able to cope and adapt in times of crisis. Special emphasis should be given to weaker populations most affected by external changes, including older people, low socioeconomic groups, those with mental health issues and minority groups, in order to reduce disparities.

## Introduction

The first cases of novel coronavirus (COVID-19) were diagnosed at the end of December 2019 in Wuhan, China. Demonstrating a rapid global spread, a pandemic was declared by the World Health Organization on March 11th, 2020. During the first 12 months, 191 countries were affected, over 77 million confirmed cases and 1.7 million deaths were reported globally [1]. Although most infected patients experience mild or moderate disease course and good prognosis, 14% of patients experience a severe course, requiring hospitalization, with case fatality rates ranging from 0.8 to 2.2% of all confirmed cases [2]. Poor prognosis characterizes mainly elderly patients and those with co-morbidities, including hypertension or diabetes. Patients aged 75–84, for example, have an 8-fold higher risk of hospitalization compared with 18–29 year olds [3]. More deprived and lower socioeconomic status (SES) communities, as well as ethnic minorities, have demonstrated higher infection rates, more severe disease and higher mortality rates [4].

In December 2020, the vaccines of two pharmaceutical companies were approved for emergency use by the US, British, Canadian and European Union regulatory authorities [5, 6]. However, many health professionals believe that public health measures, which were the main defense mechanism during the first 12 months of the pandemic, are likely to remain in place throughout 2021. These measures include social distancing, face masks and hand washing and are aimed at reducing viral spread [7]. Certain countries imposed partial or total lockdowns (“stay at home” orders) for shorter or longer periods. This contributed to the emergence of “collateral effects” of the pandemic, including avoiding seeking medical care for acute, life threatening conditions [8], and a host of mental issues associated with social isolation [9]. Beyond the direct health effects of the novel coronavirus, i.e. morbidity, hospitalization and mortality, indirect health effects were caused by lockdowns which led to drastic social and economic changes, creating mass unemployment and social isolation, which in turn affect health outcomes [10]. All of the above contributed to the direct and indirect effects of the pandemic on increased mortality rate [11].

Alongside alarming reports on the death toll, new anecdotal reports of decreased mortality began accumulating as early as 2 months from the beginning of the pandemic. For example, reductions in air pollution were reported during lockdowns in Korea and the UK due to decreased emissions from factories and vehicles [12, 13]. Other factors mentioned as contributing to decreased mortality include a decrease in car accidents [14], work related accidents [15], surgery related complications, hospital-acquired infections [16], and fewer non-COVID respiratory infections like influenza [17–19] .

Some countries showed excess mortality from the start of the pandemic while others did not, and some showed later evidence in subsequent waves. Pooled European data from the first 18 weeks of the pandemic demonstrated excess all-cause mortality compared with previous years. Some of this mortality is attributed to COVID-19 [20]. However data in some countries (including Israel, Iceland and Norway) showed no or very little excess mortality during the first 6 months of 2020, compared with previous years [21], indicating that while COVID increased the death rate, the unplanned consequences of the pandemic led to a reduced death rate from other causes in some countries [22]. In Israel, significant excess mortality was subsequently documented in August–October 2020, compared with the average of 2017–2019, particularly in the Arab sector [23]. Other countries, like the UK, Spain and Italy, showed high excess mortality [24].

Not everyone is affected equally by the changing reality of a health crisis or epidemic [25]. Some hypotheses have attempted to explain the higher COVID burden in lower SES and minority groups, looking at parallel higher comorbidity and poorer health profile, more obesity and smoking. The salutogenic model of health may help shed light on these disparities, which have been exacerbated by the current health crisis. This model proposes that people have resistance resources and deficits which lead to either deterioration of health or better health (salutogenesis) via sense of coherence and resilience building [26]. In the current study we propose a theoretical model to explain these contrasting influences on individuals’ risk of mortality during the pandemic and elucidate the underlying mechanisms involved, inspired by certain elements of the salutogenic model of resilience.

## Method

### Development of the model

The theoretical model is designed to enable a better understanding of the components, the relationship between them and their contribution to the outcome – individual mortality risk during times of health crisis.

**Fig. 1:**
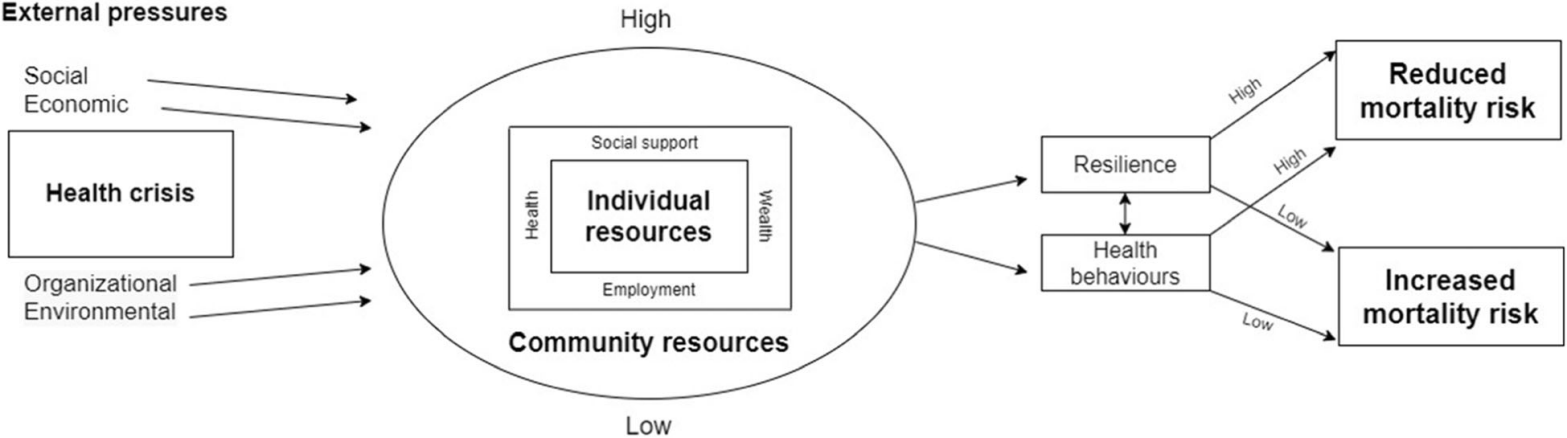
A theoretical model describing the factors influencing mortality during a health crisis

The model (Fig. 1) is composed of three components: external pressures, individual & community resources and outcomes.

### Part a: external pressures

At a time of great upheaval such as the present pandemic, numerous external pressures or changes exert influence on individuals and communities, affecting their behavior and way of life, and ultimately health outcomes and survival chances. These external pressures can include Environmental factors (e.g. changes in pollution levels, traffic, climate, access to green space), Economic factors (e.g. high unemployment rate, government handouts, failing economy), Political/Organizational factors (e.g. restrictions on movement, lockdowns, instability) and Social factors (e.g. social distancing, isolation, sense of community). These factors may influence direct health effects such as the chance of being exposed to and getting sick with COVID-19 (manual employment, travelling on public transport); as well as indirect health effects (mental health effect of lockdowns, social isolation, poorer control of chronic disease).

### Part B: individual and community vulnerability

Individual resources that a person possesses include wealth (including finances, property, savings); health (both physical and mental; comorbidities); employment (stability, income, job satisfaction, flexibility - the latter being particularly relevant to the current pandemic, manifested for example as the ability to work remotely); and social support (both practical and emotional). These resources determine the individual’s resilience and ability to adapt to change.

The resources of the community in which the individual lives further influence resilience – the existence or lack of social capital, neighborhood safety, community centers, investment in education, green spaces, sports facilities, transport networks and crime rates. High individual and community resources increase the ability to adapt, and reduce mortality risk associated with the major event like the current pandemic; while low resources reduce the ability to adapt and increase mortality risk. Furthermore, individual and community resources affect health behaviors, which in turn are associated with health outcomes, for example unemployment may lead to poorer nutrition and less physical activity, and social isolation may lead to depression or increased risk behaviors such as alcohol consumption, smoking, narcotic use or unhealthy eating. High individual and community resources may increase opportunities for positive health behaviors such as more free time to do sport, reduced work pressure, more family time due to working from home as well as increasing sense of control over events. Low resources may decrease opportunities for positive health behaviors and reduce sense of control. Available resources may also affect use of and access to healthcare, whether financial or logistical access, including help with adapting to online services.

### Resilience

According to the salutogenic model of health, a person’s confidence that they have the resources to cope with change affects their health [26]. This confidence, sometimes called sense of coherence, can provide resilience against disease and has been linked both with health behaviors and disease outcomes [27, 28]. In the context of COVID-19, high individual and community resources might increase resilience and the ability to adapt to external pressures and change, thereby reducing mortality risk [29]. In contrast, low resources might reduce resilience and the ability to adapt, increasing mortality risk.

#### Increased mortality rate

Alongside deaths caused directly by COVID-19, there are rising concerns due to the dramatic decline in health care utilization leading to delayed diagnosis of disease, including acute life-threatening conditions. In India, poor residents of deprived neighborhoods had difficulty accessing healthcare for non-COVID conditions during lockdowns [30]. In Israel, populations at higher risk, such as breast cancer patients, reported less contact with health care professionals during April 2020, the time of the first peak of pandemic spread [31].

Furthermore, despite increased availability of telemedicine services, patients who are not technologically proficient may be at greater risk of missing out on essential care. Patients with fewer resources, including older patients, those with a language barrier (immigrants), or without internet access, will have more obstacles to accessing telemedicine. Indeed, in a study of rheumatologists from around the world, while most had switched to remote appointments, 17% reported that around a quarter of their patients did not have access to telehealth video, especially those from below the poverty line [32]. It has been demonstrated that social networks influence individuals’ adaptation to new technologies in organizations [33]. Older senior citizens (70+) have been found to use the internet less frequently. The increasing number of public and private services that are re-designed as online solutions, and the emergence of new applications, further excludes those seniors, and others with low digital literacy, from active participation [34]. In times of a health crisis, access to online sources of information and advice is especially important.

Additionally, physical distancing used as the main mitigation strategy during the current pandemic, especially in the first 12 months (before the emergence of safe and effective vaccines), has had a substantial impact on both the economic situation and mental health state of individuals. The prevalence of depression among US adults increased approximately three-fold [35]. Mounting experience shows that large scale events such as the current pandemic are almost always accompanied by a myriad of mental health consequences with increased rates of depression, post-traumatic stress disorders, substance abuse, domestic violence and child abuse [36]. Mental health issues deplete resources and reduce resilience. As such, there are great concerns about a significant rise in suicide rates and acute stress-related medical conditions, such as acute myocardial infarction or acute ischemic stroke—which can ultimately result in increased mortality [36]. Individuals with pre-existing mental health issues or at risk of social isolation (ie. weakened resources), start from a more precarious point, and are more likely to feel the negative effects of the changes [37, 38].

A Canadian study found that minority groups experience greater COVID-19 related mental health issues compared to non-minority groups, including depression and anxiety [39]. In a US study, individuals with lower social resources, lower economic resources and greater exposure to stressors (losing a job, death of a family member from COVID-19, experiencing financial problems) were more susceptible to depression, with lower income respondents at 2.37 times higher risk of depression [35]. A survey conducted before the pandemic demonstrated that people with low family savings had more depressive symptoms [40]. In times of health and economic crisis like the current pandemic, the absence of a financial safety net in the form of family savings, job security or property might explain the higher prevalence of depression among the weaker segments of the population.

#### Decreased mortality rate

Individuals who enjoy high individual and community resources may have been able to benefit from the change in circumstances imposed by the pandemic. Mitigation strategies implemented across the world have included temporary lockdowns for a majority of the population, and social restrictions, with continued employment allowed solely for those considered essential workers. Less traffic has led to lower rates of car accidents (a decrease of ~ 20%) [41]. work-related accidents (~ 70%) [15] and air pollution (~ 30%) [12, 13]. Influenza cases reduced dramatically, with hardly any noted cases in some countries in comparison to high rates of flu morbidity in previous years [19].

Imposed restrictions initiated by the pandemic may also have had a positive influence. Transitions to remote working from home may reduce stress related conditions often associated with hectic working and commuting routines, among those with the ability and circumstances to do so. However, remote working is only possible for those in certain jobs, generally more stable and better paid professions, while individuals in physical or menial jobs, often more precarious to begin with, do not reap this benefit. A strong social support network is also likely to help in adapting to the new reality.

## Discussion

The COVID-19 pandemic has impacted the entire world, causing a global health challenge, and an escalating number of deaths. The pandemic has affected every sphere of daily life, influencing health, social life, employment and environment. While in some countries significant excess mortality has been reported, in others, excess mortality was not demonstrated in 2020, with COVID deaths compensated for by decreased mortality in other spheres [41]. While evidence of this secondary positive impact is encouraging, the worrying fact is that disadvantaged groups, with lower resources at their disposal are likely to bear the brunt of the pandemic. Minority and low SES groups have been shown to have both greater risk of infection [42], as well as higher mortality rates [43].

Sociodemographic characteristics affect the impact of COVID on individual behaviors, whether in a positive or negative direction, with the most disadvantaged members of society – with less resources and resilience - potentially suffering more, as has been demonstrated in some countries. A simulation study using data from the US, China, UK, Spain, Italy and France found associations between social determinants of health and COVID-19 mortality, similar to the level of association between hypertension/diabetes and COVID-19 mortality [43], indicating that people from low-income and low-education groups will require additional support.

One notable example is the move to home working arrangements for many businesses, which has led to a significant reduction in air pollution and a reduction in the rate of car accidents [13], however this arrangement is only available to a proportion of workers, leaving those with more precarious menial jobs at an even greater disadvantage. Furthermore, individuals with low digital literacy are left behind in the current pandemic in terms of social support, receiving information and accessing resources which have all transferred to online versions. This is also true for home schooling, which is dependent on resources, indeed an OECD study found that low-income and/or single-parent families were likely to be the most affected by the closure of schools and childcare facilities and transition to digital learning [44].

Long term social, economic and health impacts are expected to comprise the largest challenge in the recovery from the pandemic. The pain and suffering of the pandemic are not equally borne, with the circumstances imposing disproportionate risk and impact based on structured ethnic, class and occupational inequalities. This inequality has been summed up during the current pandemic thus: “We are all in the same storm, but we are not all in the same boat,” [45, 46].

The economic and social implications of the pandemic are still only partially understood. However, it is clear that job losses are greater for ethnic minorities and less educated individuals [47]. Vulnerable populations are over-represented in occupations that require more interpersonal contact and cannot be performed remotely, and are therefore both at greater risk of exposure, and more prone to unemployment, with all its negative consequences [4, 47].

Despite the positive influence of decreased accidents, air pollution and non-COVID respiratory infections, quarantine and social isolation is expected to increase the rate and severity of mental illness and suicides. This is also likely to disproportionately affect individuals with low resources and resilience. For example in the South Korean economic crisis of 1997, suicides were more prevalent in those with lower educational level [48].

While decisions about lockdowns were based largely on the direct health impact of COVID-19, the proposed model might help policymakers, healthcare professionals and researchers to have a more holistic perception of the consequences of the pandemic and invest in community resources to help people weather change. Special attention should be paid to the vulnerable segments of the population, including both those with low SES, but also those with weaker social networks or mental health issues, where most preventive measures should be directed to reduce the health consequences of this crisis. The OECD proposed a resilience-based approach to management of epidemics, focusing on the ability of systems to adapt and recover, indeed systems should be adapted to help those least resilient to change [44].

While developed in relation to the COVID-19 pandemic, the model is also relevant to the impact of other major events, including war, natural disaster and economic depression. The external pressures may vary, but the mechanism and essential effect of internal and external resources remain the same.

As the long-term effects of the pandemic are likely to stay for some years, decision makers should consider diverting the resources gained from decreased mortality in some spheres to better support vulnerable populations during the pandemic and as part of the exit strategy.

## Conclusion

By shedding light on the underlying mechanisms and human behaviors that influence the outcomes of a health disaster, the model can help to direct efforts to the most vulnerable populations, thus improving preparedness across the board. Beyond the known social gradient in health, the model proposes other factors, including social, psychological and community resources which may affect the extent to which individuals are affected by the pandemic. Based on the lessons learned from the theoretical model, it is recommended to define a body responsible for identifying and caring for populations at risk during a health disaster. Such a body would examine how to prevent or at least reduce the widening of disparities, in order to tip the scales towards lower morbidity and mortality.

The recent start of the vaccination program which should eventually dramatically decrease viral spread, means the light at the end of the tunnel is beginning to emerge. However, given limited initial supplies of the vaccine, and unequal distribution around the world, it might still take many months before a significant proportion of the world is vaccinated. Looking beyond the pandemic, societies should invest in building resources to improve both individual and community resilience to help people be better prepared and more able to cope and adapt in times of crisis.

## Data Availability

All data available in the Ministry of health, Israel website- URL: https://govextra.gov.il/ministry-of-health/corona/corona-virus-en/

## Abbreviation

COVID-19: Corona Virus December 2019
